# Mechanical-Chemical Combined Atherectomy in a Topologically Isolated Arterial Segment

**DOI:** 10.7759/cureus.109869

**Published:** 2026-05-29

**Authors:** Ilia Toli

**Affiliations:** 1 Materials Science and Engineering, University of Texas at Dallas, Richardson, USA

**Keywords:** atherectomy, atherosclerosis, balloon catheter, biodegradable coating, extracorporeal circulation, interventional cardiology, mechanical-chemical atherectomy, percutaneous arterial access, plaque regression, topological isolation

## Abstract

This work extends a closed-loop extracorporeal vascular cleaning architecture, introduced in a prior publication, with four refinements that address practical and safety considerations not handled in the prior protocol. First, atheroma is hypothesized to consist of two mechanically distinct fractions: a weakly adhered fraction removable by gentle mechanical contact within the isolated chamber, and a structurally integrated fraction requiring chemical action. Mechanical pre-cleaning with a pointed balloon under inline pressure feedback addresses the weakly adhered fraction first; chemistry then operates against an exposed substrate. The two-regime model bounds the residual chemistry burden at the structurally integrated mass fraction, hypothesized at 40%-60% of total plaque mass and corresponding to an upper bound of 60% of the chemistry-only collagenase specification; exposed-substrate kinetics may permit further reduction, though the magnitude of any such enhancement is undefended at the concept stage and must be established empirically. Second, the pressure differential between the bypass and treatment circuits is made stage-dependent: inward-biased during chemistry and outward-biased during saline phases, with a bounded outward sweep during the final flush. Third, direct percutaneous needle access through the overlying tissue extends the architecture to deep vessels (renal, mesenteric, and retroperitoneal) currently inaccessible to catheter-based intervention. Fourth, a localized biodegradable protective coating applied to the cleaned vessel wall before blood reintroduction bridges the re-endothelialization window with local protection rather than systemic antithrombotic therapy. Each refinement uses approved or near-approved components in new combinations. Two architectural extensions are also developed: a hemodynamic synergy with systemic therapy in which cleaned segments serve as preferential redeposition sites for material mobilized from inaccessible regions, and an organ as chamber primitive generalizing topological isolation to organs with discrete arterial inflow and venous outflow. Five testable predictions are specified for validation in a porcine atherosclerosis model.

## Introduction

The closed-loop extracorporeal vascular cleaning architecture introduced in a study by Toli established a topological isolation primitive for chemical atherectomy [1]. The present work develops what the chamber additionally enables.

In the architecture of Toli, two balloon catheters bracket the target lesion; the segment between them is drained of blood and connected to an external bypass that maintains distal perfusion [1]. The treatment zone becomes a sealed saline chamber in which chemical agents (unable to be deployed in flowing blood at therapeutic concentrations) dissolve the major components of atherosclerotic plaque at their respective vulnerabilities: ethylenediaminetetraacetic acid (EDTA) for calcified deposits, surfactants for the lipid-rich necrotic core, and collagenase for the fibrous cap.

The chemistry-only protocol works against the entire plaque structure as a single chemical target. In practice, atheromatous material is heterogeneous in its adhesion to the vessel wall: some fraction is weakly held and would yield to gentle mechanical contact, while another fraction is structurally integrated and requires chemical degradation. Treating both fractions identically with the same chemistry burden overdoses the weakly adhered fraction and reaches the structurally integrated fraction only after removing the overlying material chemically.

The chamber removes the constraint that has historically prevented mechanical-chemical combined approaches. In flowing blood, mechanical disruption generates embolic debris that endangers downstream tissue, which forces chemistry-only or mechanical-only approaches but not their combination. With blood removed, debris flushes out through the effluent circuit, mechanical tools previously unsafe become deployable, and chemistry and mechanics can be combined in a single session.

The paper's primary audience is the interventional vascular community: cardiologists, vascular surgeons, and interventional radiologists who perform or develop atherectomy procedures, and the technical sections are calibrated accordingly. The architecture's reach into adjacent specialties is nevertheless worth flagging at the outset: renal arteries (nephrology), mesenteric and visceral vessels (gastroenterology and hepatobiliary medicine), pudendal arteries (urology), the parallel organ as chamber primitive applicable to discrete vasculature organs (urology, endocrinology, and transplant medicine), biodegradable coatings (biomaterials engineering), and the hemodynamic redistribution interaction with statin and proprotein convertase subtilisin/kexin type 9 (PCSK9)-driven therapy (lipidology). Readers from adjacent specialties may find the introduction, the two-regime plaque model, the discussion of synergy with systemic therapy, the broader needle access and organ as chamber subsections, and the conclusions sufficient to grasp the architecture; the technical subsections on mechanical tools, pressure regimes, coating chemistry, and validation detail may be treated as supporting material on a first reading.

The paper develops the combined protocol -- supported by the pointed balloon with pressure feedback tool and existing atherectomy methods reconsidered within the chamber -- and four refinements: a two-regime plaque model, stage-dependent pressure as a safety regime, direct percutaneous needle access for deep vessels, and a localized biodegradable protective coating to bridge the re-endothelialization window. Two anatomical extensions, the coronary ostia and the aortic valve, are recorded as future directions. Testable predictions and limitations follow.

## Technical report

Two regimes of plaque material

The combined protocol rests on a two-regime model of plaque material. Atheromatous plaque, taken as a class, has two mechanically distinct fractions: a weakly adhered fraction that is loosely held to the underlying tissue and would yield to gentle mechanical contact within the isolated chamber, and a structurally integrated fraction that is bonded into the matrix at a level requiring chemical degradation rather than mechanical removal. The first fraction includes surface lipid deposits, loose foam cells, accumulated soft material, and recently deposited sludge that has not yet been crosslinked into a stable structure. The second fraction includes mature fibrous cap collagen with established matrix integration, calcified deposits embedded in surrounding matrix, and cholesterol crystals locked into oxidized lipid networks. The boundary between the two is empirical and varies by lesion morphology, age, and treatment history.

The working hypothesis is that the weakly adhered fraction accounts for an estimated 40%-60% of total plaque mass on a typical lesion. This range is a hypothesis, not a measured quantity: published data anchor compositional heterogeneity within plaques (lipid core, fibrous cap, and calcified deposits) [2,3] and document wide variation in plaque morphology across lesion populations [4], but adhesion strength as such has not been measured at scale. Mechanical debulking outcomes from existing atherectomy devices yield 30%-60% volume reduction [5]; this measures what the device removes at its embolic-conservative operating settings, which is jointly determined by adhesion and applied force, not adhesion alone. The two-regime model is itself a projection of a higher-dimensional substrate space, in which adhesion strength, chemical susceptibility, and depth of integration are not equivalent partitions; a calcified nodule may be mechanically weakly attached but chemically resistant, or mechanically integrated but chemically accessible. The mechanical-first ordering is robust to this projection error: weakly attached material leaves during the mechanical pre-cleaning stage regardless of its chemical susceptibility, and integrated but chemically accessible material is left for the chemistry stage, where its accessibility is exactly what is needed. In the off-diagonal cases, the simple binary misses are handled correctly by the protocol's sequencing, not in spite of it. Quantifying the actual partition across a representative population is a testable prediction 1.

The protocol in a study by Toli treats both regimes through chemistry, applying enzymatic and chelating agents against material that would yield to gentle mechanical contact [1]. The mechanical-only approaches (rotational, orbital, laser, and directional atherectomy) treat both regimes through mechanical action, attempting mechanical disruption against material that resists at any safe contact pressure. Combining them in sequence (mechanical first, chemistry second) matches the treatment to the substrate.

Pointed balloon with pressure feedback

The primary mechanical tool for the weakly adhered fraction is an angioplasty balloon catheter modified with two features: a tapered or pointed leading edge (1-2 mm) that advances through partially occluded segments more easily than a blunt cylinder, and an inline pressure sensor that monitors advancement force. The body of the balloon remains cylindrical for full circumference contact during cleaning. Inflation pressure can be varied along the segment as the balloon advances, with lower pressure across narrower or more eccentric regions and higher pressure across regions where the lumen is more open or the operator wishes to apply more contact force; this is straightforward with both compliant and non-compliant balloon designs.

Why a modified balloon rather than a novel tool

Angioplasty balloons are FDA-approved at scale, manufactured by multiple vendors, familiar to every interventional cardiologist, and have well-characterized failure modes. Inline pressure sensors are standard components in catheter handles for inflation monitoring; extending them to advanced pressure monitoring is a small modification, not a new technology integration. The regulatory path is dramatically shorter than for any novel device design (rotational burr, dedicated brush, and ultrasonic probe), shortening time to clinic by an estimated 5-10 years. Full circumference contact is intrinsic to a fully inflated balloon, eliminating concerns about eccentric plaques deflecting the tool, and longitudinal advancement requires no new procedural training.

Why pressure feedback rather than visual imaging

The advancement pressure signal is a single number, measured directly by a transducer in the catheter handle, with no optical components, image processing, or display interpretation required. It operationalizes the cleaning decision as a threshold rule (stop when force exceeds X), removing operator variability in a way that visual interpretation cannot. It also captures information a camera cannot: a small but hard, calcified nodule and a large but soft lipid deposit may look similar visually but require different mechanical responses, and pressure feedback distinguishes them by mechanical resistance directly.

Imaging remains an option for later refinement. Saline is acoustically and optically simpler than blood, so intravascular ultrasound (IVUS), optical coherence tomography (OCT), and near-infrared spectroscopy all work better in the chamber than in flowing blood. They are available where visual confirmation is needed but are not required for the basic protocol.

Calibration cycle

The patient-specific safe threshold is unknown a priori, which makes the first cycle in any new vessel exploratory. The protocol specifies an explicit calibration cycle before the first cleaning pass, with the operating threshold initialized at a conservative population-derived ceiling and adjusted only downward, never upward, on the basis of resistance events encountered. Specifically, the balloon advances at the population-ceiling threshold; if any non-zero resistance event is detected, the balloon stops, deflates, and the operating threshold for that vessel is reset to a safety factor multiple (typical safety factor: 0.6-0.8) of the resistance value at that point. The threshold thereafter only ratchets down further if subsequent resistance events occur at lower values. The asymmetry matters: the first detected resistance may be plaque or may be wall, and the operator does not know which. Treating it as wall by default and adjusting the threshold downward ensures that misidentification of plaque as wall costs a slightly conservative cleaning, while misidentification of wall as plaque (which an upward adjustment would permit) costs medial damage. The calibration cycle occupies at most one minute and converts an unknown into a measured upper bound before any aggressive advancement.

Retrograde saline unjamming

When the balloon stops at threshold, the operator may attempt a retrograde saline pulse: a brief reversal of the treatment circuit flow direction that injects saline into the lumen distal to the balloon, applying hydraulic force in the direction opposite to balloon advancement. This unjams loose material that may have wedged against the balloon's leading edge without engaging the underlying vessel wall. The maneuver costs seconds, leaves no residue, and is bounded in pressure by the chamber's pressure-differential safety regime. If unjamming succeeds, the balloon advances under its calibrated threshold; if it fails, the operator concludes that the resistance is from material requiring chemistry rather than mechanical action and proceeds to the next stage.

Spatial mapping of plaque distribution

Each position where the pressure exceeded the threshold is a location with significant mechanical resistance, likely a calcified or fibrous plaque region. Each position where the balloon advanced freely is a clean or soft plaque region. The pressure log per cycle, combined with the effluent assays per cycle, generates a two-channel spatial profile of the entire treated segment: a mechanical resistance map and a chemical composition map.

The resolution of this method has limits worth stating. Advancement pressure integrates resistance over the full balloon-vessel contact area, so the signal is a low-pass filter on the underlying mechanical profile. A small focal hard nodule embedded in soft material may not raise the integrated signal above threshold even though the local contact pressure on the nodule itself is high. Axial localization (along the vessel) is good because the balloon advances by discrete increments, and each increment maps to one pressure value. Circumferential localization is poor because the balloon contacts the entire vessel circumference simultaneously. Sub-balloon-length localization is also poor because the integrated signal does not distinguish a single hard inclusion at one end of the balloon from multiple smaller inclusions distributed across its length. The safety implication is direct: a focal hard inclusion can produce extreme local stress on intima or media without the integrated pressure signal flagging it, which means the calibrated threshold must be conservative relative to the population safe contact pressure rather than aggressive, and lesions known or suspected to harbor focal calcified spikes warrant supplementary imaging within the saline chamber. For applications where focal hard inclusions matter, IVUS or OCT in the saline-filled chamber recovers the missing channels. The pressure feedback signal does the gross spatial work; imaging supplements it where the resolution limit binds.

Texture variants and tool agnostic principle

The protocol can use different balloon types within the same session, depending on what the effluent signal shows: plain balloons for the most loosely adhered material, scoring balloons for organized thrombus, and cutting balloons for severely calcified segments. The principle is implementation-independent. Inside the isolated chamber, gentle contact removes the weakly adhered fraction of plaque; the specific device that applies the contact (any of the balloon variants, a spiral wire, a brush, an ultrasonic probe, or a future design) is an engineering choice driven by availability, cost, regulatory path, and tuning requirements.

The balloon is recommended as the first-generation implementation because existing approval, manufacturing, and clinical familiarity make it the fastest path to the clinic. This is the standard pattern in interventional cardiology: Grüntzig's founding paper introduced a specific balloon, and the principle has since accommodated drug-eluting, scoring, and cutting variants developed decades later. The principle is what the literature cites; specific instruments evolve.

Existing atherectomy methods reconsidered within the isolated chamber

The recommendation of a modified balloon as the first-generation mechanical tool should not be read as a claim that other mechanical methods are inappropriate within the isolated chamber. They are not just appropriate; they are enhanced by it. Every existing percutaneous atherectomy method has a wider operational envelope inside the chamber than it does in flowing blood because the constraint that has governed the field for four decades is transformed: distal embolic risk is eliminated, and what remains is a local damage budget against the vessel wall. The constraint is not gone, but it has shifted from a downstream-circulation problem to a local-tissue problem, and local-tissue tolerance is a quantity an operator can monitor and bound in real time in a way that distal embolic events cannot be intercepted after they occur. This section addresses each major method in turn within this transformed envelope, then makes the combinability argument.

The constraint existing methods were designed around

The fundamental constraint on every existing atherectomy method (rotational using the Rotablator, orbital using the Diamondback 360, excimer laser, directional using the SilverHawk, and others) is that liberated debris enters the bloodstream and must either be small enough to clear safely through the microcirculation or be captured by distal embolic protection devices that work imperfectly [6]. This constraint forces conservative operational settings: lower rotational speeds, shorter ablation pulses, smaller cutting bites, and partial rather than complete plaque removal. Aggressive removal is unsafe even when the method is mechanically capable of it.

Rotational and orbital atherectomy within the chamber

Rotational atherectomy uses a diamond-coated burr rotating at 140,000-190,000 rpm to abrade calcified plaque into particles roughly the size of red blood cells, on the assumption that such particles will pass through the microcirculation without infarcting end organs. In the chamber, this assumption is unnecessary: the burr can run at any speed, generate particles of any size, and the operator can engage harder calcified deposits without fear of distal embolization. Treatment time per lesion segment may decrease substantially because aggressive engagement no longer requires sequential gentle passes. Orbital atherectomy uses an eccentric crown to grind plaque through differential sanding action; the same logic applies. Within the chamber, the orbital amplitude can be larger and the contact more sustained.

Excimer laser atherectomy within the chamber

Excimer laser atherectomy ablates plaque through ultraviolet pulse energy that vaporizes tissue and creates a plume of microbubbles and microparticulate debris. The conventional concern is that the vapor and microparticles enter circulation; in the chamber, the plume is captured in the saline effluent and filtered out. The laser energy can be increased and the pulse rate raised because there is no embolic consequence to more aggressive ablation. Vessels heavily calcified beyond the practical reach of conventional rotational atherectomy become accessible.

Directional atherectomy within the chamber

Directional atherectomy excises plaque as discrete shavings collected in a nose-cone reservoir. The conventional limit is the reservoir capacity: when full, the catheter must be withdrawn, emptied, and reinserted. In the chamber, the shavings can be flushed through the effluent circuit instead of accumulating in a fixed reservoir, allowing continuous operation through a complete lesion in a single pass.

Other methods

Ultrasonic ablation uses high-frequency mechanical vibration to disrupt plaque through cavitation effects. Conventional applications in flowing blood are limited because cavitation in blood produces complex bubble dynamics, and the disrupted material has to clear safely downstream. In saline within the chamber, cavitation is cleaner and more controllable, and the disrupted material flushes out in the effluent. High-pressure water jet systems, methods that rely on chemical-mechanical hybrid action, and tools yet to be developed all face the same enabling environment within the chamber.

Combinability and method switching within a single session

Within the chamber, debris of any size flushes out through the effluent circuit. Nothing reaches the systemic circulation. The distal-embolic constraint that historically forced conservative settings does not apply. Each method can be operated closer to its mechanical limit, with the operator choosing settings based on tissue removal efficacy and local damage tolerance rather than embolization risk. The methods that exist today acquire a wider operational envelope without hardware change.

The combinability argument extends further. Different mechanical methods are optimal against different plaque substrates: rotational atherectomy excels at hard calcified deposits, directional atherectomy at focal eccentric lesions, laser at fibrocalcific mixtures, and balloons at distributed soft material. In conventional practice, switching between methods within a single session is rare because each method change requires a new device exchange, with the embolic exposure of debris already generated, plus the time and risk cost of additional sheath exchanges. Within the chamber, the embolic transition cost is removed. The mechanical transition cost (sheath manipulation, sequential device exchange, and repositioning) remains and adds to total session time the same way it does in conventional procedures. What the chamber changes is that switching between methods no longer requires a debris-clearing wait period or distal-protection redeployment between exchanges, and the safety penalty for mid-session method switching collapses. A treatment session can begin with a balloon pass to remove the weakly adhered fraction, continue with rotational atherectomy on the exposed calcified deposits, switch to laser ablation for fibrocalcific lesions resistant to mechanical disruption, and finish with directional atherectomy of focal residuals, all within a single chamber session, with debris flushed continuously through the effluent circuit and all per-method operating settings chosen for maximum efficacy rather than minimum embolic risk. Total session time remains constrained by ischemic tolerance of territories supplied by minor branches within the isolated segment, as discussed in the combined protocol section.

Implications for practice and device industry

The argument has practical consequences. A device industry that has spent four decades optimizing for the embolic debris constraint may find that constraint transformed within the chamber architecture, and that the operating parameters of every existing percutaneous atherectomy device deserve re-evaluation in this new environment. This re-evaluation does not require new devices. Existing FDA-approved equipment, used at parameter settings appropriate to the chamber, is sufficient to begin clinical investigation. The first chamber-based clinical trials may use an off-the-shelf rotational atherectomy device at a higher rotational speed than its current label specifies, an off-the-shelf laser at higher pulse energy, or a standard balloon under the pressure-feedback protocol. None of these requires a new 510(k) submission for the device itself; they require an investigational device exemption for the chamber application, which is a different regulatory pathway with substantially shorter typical timelines.

Combined mechanical-chemical protocol

The combined protocol sequences mechanical pre-cleaning followed by reduced chemistry within the same isolated chamber. Table 1 summarizes the stages, methods, plaque targets, completion endpoints, and approximate per-stage durations. The order presented in the table is one reasonable default; the architecture does not impose a fixed sequence, and the operator may reorder or repeat stages based on what each stage's effluent signal reveals about the lesion.

**Table 1:** Combined mechanical-chemical protocol stages, methods, plaque targets, completion endpoints, and approximate per-stage durations EDTA, ethylenediaminetetraacetic acid.

Stage	Method	Target	Endpoint	Time
0. Initial saline rinse	Bidirectional saline at a moderate flow rate	Loose, non-adherent debris and surface material	Effluent visibly clear; turbidity at baseline	2-5 min
1. Mechanical pre-cleaning	Pointed balloon, pressure-feedback advancement, retrograde unjamming	Weakly adhered fraction (40%-60% of plaque mass)	Pressure log clean; effluent debris assay returns to baseline	10-20 min
2. EDTA	Disodium EDTA 50-100 mM, bidirectional flow	Hydroxyapatite (calcified deposits)	Effluent Ca²⁺ returns to baseline (corrected for sentinel-detected blood admixture)	5-10 min
3. Surfactant	Poloxamer 188 1%-5% w/v or HP-β-CD 10-50 mM, bidirectional flow	Lipid-rich core (residual after mechanical)	Effluent turbidity and lipid colorimetry return to baseline	5-10 min
4. Reduced collagenase	0.05-0.25 mg/mL (≤60% of chemistry-only dose, hypothesized)	Structurally integrated fibrous cap	Effluent hydroxyproline returns to baseline	3-5 min
5. Final saline flush	Heparin-bonded saline, bidirectional; outward pressurized at 1.2-1.5× bypass for ≤30 s	All residual chemistry; antithrombotic surface coating	Effluent channels within 2σ of pre-procedure baseline, sustained ≥3 cycles	5 min
6. Biodegradable coating (optional)	In-situ polymerization in a saline-filled chamber	Re-endothelialization window protection	Coating fully formed; visible by post-chamber imaging	2-5 min

A note on the chemistry's prior history is warranted before the protocol detail. EDTA chelation has a clinical record in cardiovascular disease as a systemic infusion therapy, most prominently in the Trial to Assess Chelation Therapy (TACT) and TACT2 trials [7]. The signal in those trials was modest and contested, and the natural reader objection is: if systemic EDTA infusion shows only a weak effect on cardiovascular endpoints, why should chamber-isolated EDTA be expected to dissolve plaque calcium effectively? The answer is concentration. Systemic EDTA infusions reach plasma concentrations on the order of 1 mM at peak, distributed across the entire circulating volume, and constrained by renal toxicity at higher exposures. Chamber-isolated EDTA at 50-100 mM, locally confined to a small treatment volume and with no systemic exposure, is a different physical regime. Published hydroxyapatite dissolution rates in EDTA scale approximately linearly with chelator concentration in the relevant range [8], implying order-of-magnitude faster surface kinetics in the chamber regime than under systemic conditions. The TACT data establish that systemic EDTA at tolerable doses has a weak local effect; they do not establish that local EDTA at orders-of-magnitude higher concentration will be similarly weak. The chamber concentration regime is the relevant test, and that test has not been performed.

The dissolution argument has a transport-limited component that the concentration argument alone does not address. EDTA must reach hydroxyapatite crystal surfaces through the surrounding plaque matrix; matrix permeability, competing-ion equilibria (notably Ca²⁺ buildup in the immediate vicinity of dissolution), and the time available within an ischemic window all bound the achievable dissolution rate. The mechanical pre-cleaning stage of the combined protocol mitigates the transport limit by removing overlying material and exposing crystal surfaces directly to the chamber medium. This is the kinetic basis for the claim that chemistry against an exposed substrate proceeds faster than chemistry against a buried substrate; the specific rate enhancement is empirical and is part of what the porcine validation in testable prediction 3 must measure.

**Figure 1:**
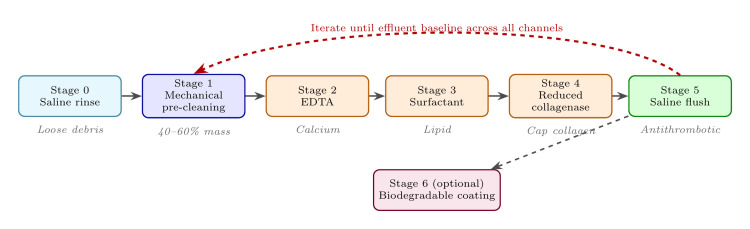
Stages of the combined mechanical-chemical atherectomy protocol within a topologically isolated arterial segment Figure created with TikZ/PGF (LaTeX) EDTA, ethylenediaminetetraacetic acid.

The per-stage detail proceeds as follows. Stage 0, the initial saline rinse, precedes all active agents: a moderate-flow bidirectional saline rinse clears loose, non-adherent debris from the chamber. This step is inexpensive in time (a few minutes) and material (saline). The volume of debris recovered varies widely by lesion: lesions with a thick layer of recent thrombus, accumulated sludge, or already loose surface material may yield substantially in this stage alone, reducing the workload of the mechanical and chemistry stages that follow. Stage 1, mechanical pre-cleaning, applies the balloon protocol described above to remove the weakly adhered fraction: surface lipid deposits, loose foam cells, soft material, and accumulated sludge that survived the initial saline rinse, estimated at 40%-60% of total plaque mass on a typical lesion. Stage 2, the EDTA stage, follows the protocol in Toli and now operates against an exposed substrate rather than buried calcified nodules [1]. The mechanical step has stripped the overlying soft material that previously made calcium hard to reach; dissolution rates accelerate substantially because the surface area exposed to the chelator is much larger. Stage 3, the surfactant stage, also follows the protocol in Toli: buried lipid in foam cells is now closer to the saline interface, and emulsification proceeds more efficiently [1]. Stage 4, the reduced-dose collagenase stage, uses a scaled-down version of the protocol in Toli [1]. The most easily removable fibrous material is gone via mechanical action. Remaining collagen is the structurally integrated portion that needs enzymatic cleavage anyway, but the dose and exposure time can be substantially shorter than in the chemistry-only protocol. The two-regime model alone bounds the residual chemistry burden at ≤60% of the chemistry-only burden, corresponding to the structurally integrated mass fraction. Exposed-substrate kinetics may provide an additional efficiency multiplier, but the magnitude of any such enhancement is undefended at the concept stage; the actual residual must be measured empirically (testable prediction 3). Stage 5, the final saline flush, follows the protocol in Toli: heparin-bonded rinse, blood readmission, balloon deflation, and catheter withdrawal [1]. The full sequence is shown graphically in Figure 1.

The collagenase reduction is the most important practical consequence. The chemistry-only protocol's main safety concern was collagenase immunogenicity over repeated sessions: collagenase from *Clostridium histolyticum* is immunogenic, and patients who develop sensitization in early sessions cannot receive it in subsequent ones. With mechanical pre-cleaning, dose and exposure time both drop, and sensitization risk over multiple sessions becomes more manageable. Patients excluded from later sessions in the chemistry-only protocol may continue in the combined protocol.

Flow direction and stage order are free parameters

Two procedural choices that the protocol in a study by Toli fixed are open in the combined protocol [1]. First, the direction of flushing is arbitrary at any stage. Saline and chemical agents can flow proximal-to-distal, distal-to-proximal, or alternate within a single stage; the chamber is symmetric, and nothing about the architecture privileges one direction. The bidirectional flow regime in stages 2 and 3 is the default; an operator who observes uneven effluent signals from the two directions can reweight as needed.

Second, the stage order is operator-dependent. The default in Table 1 reflects the substrate-cohesion logic of mechanical first, then chemistry, with calcium dissolution before lipid emulsification, before fibrous cap digestion within the chemistry block. The architecture does not enforce this. An operator who observes (from the initial saline rinse or from imaging) that a particular lesion is dominated by surface lipid may run the surfactant stage first; one who observes a heavily calcified plaque may reorder to attack calcium before the mechanical step. Stages may be repeated. The protocol's structure is a set of available stages, not a fixed pipeline.

The chemistry and mechanical steps support each other bidirectionally: chemistry softens substrates so mechanical removal becomes effective on resistant material, and mechanical removal exposes deeper material that the next chemistry pass can reach. A cycled sequence (mechanical, EDTA, mechanical, surfactant, mechanical, and collagenase) is therefore one operator option among several. Whether to cycle, and how many times, is a choice each operator makes against the time budget of the specific session, the ischemic tolerance of the territory, and the per-stage effluent signals as they accumulate.

General parameter freedom within the chamber

The freedom of stage order and flow direction generalizes to other operational parameters that are coupled in flowing blood interventions but decoupled within the chamber. Chemistry concentration, treatment temperature, flow rate, stage duration, pressure differential, and other axes are independent free parameters, each tunable to optimize for the rate-limiting constraint of the specific lesion and patient. Hypothermia of the treatment circuit, for instance, can extend ischemic tolerance for territories with poor collateral perfusion (renal and mesenteric) without sacrificing chemistry kinetics, since chemistry concentration can be increased independently to restore reaction rate; the systemic toxicity ceiling that bounds concentration in flowing blood does not apply within the chamber, so concentration is a free axis. Warming the chamber moderately above body temperature can accelerate dissolution kinetics for lesions where total session time is the binding constraint, bounded above by endothelial thermal tolerance (approximately 42-43°C for sustained exposure). Bypass circuit temperature can be regulated independently from treatment circuit temperature, so distal tissue can be maintained at body temperature for metabolic protection, while the isolated segment runs cooler or warmer as needed. The optimal parameter combination is empirical and lesion-dependent, established through systematic validation rather than fixed a priori. The architecture supports a parameter space; the protocol specifies one trajectory through it; future researchers will map the surface.

Time

The combined protocol can run faster overall than the chemistry-only protocol despite adding the mechanical and saline rinse stages. The chemistry-only protocol's session duration was bounded below by the time required for chemistry to reach buried substrates through overlying material; with the bulk fraction already removed before chemistry runs, the per-stage chemistry exposure times drop, and the total session time across all stages can be shorter than the chemistry-only baseline. Session duration is also more predictable because the rate-limiting step is no longer an uncertain transport time through a heterogeneous matrix but a sequence of stages, each terminated by an objective effluent endpoint. A combined session that runs faster than the chemistry-only session for the same lesion is a realistic outcome and one of the practical motivations for the combined approach.

Synergy with systemic therapy and hemodynamic redistribution

The chamber architecture interacts productively with ongoing systemic therapy. Statin-driven and PCSK9-driven plaque regression, slow natural turnover of plaque components, and future targeted therapeutics all mobilize plaque material from inaccessible small vessels and diffuse deposits into the systemic lipid pool. By fluid dynamics, this mobilized material preferentially redeposits at sites of lower shear stress. Cleaned chamber-treated segments, with their restored larger lumen and consequently slower flow velocity, present lower local shear stress than they did pre-cleaning. The cleaned segments thus function as hemodynamic sinks that concentrate redistributed plaque from inaccessible regions of the vasculature, which subsequent chamber sessions can remove.

Over multiple cycles of chamber intervention coupled with continuing systemic therapy, the cumulative reduction in total body atheroma burden therefore exceeds what direct chamber extraction alone would achieve. The architecture indirectly captures plaque from regions it cannot directly reach, by exploiting the hemodynamic redistribution that systemic mobilization produces. This synergy is a feature of the architecture's clinical positioning: chamber sessions and systemic therapy are not competing modalities but complementary, with each strengthening the other across the patient's lifetime of management. The biodegradable coating refinement described in the section that follows addresses the temporary local vulnerability of the cleaned segment during re-endothelialization, after which the segment returns to its native baseline susceptibility while continuing to function as a redistribution sink for material mobilized from elsewhere.

The body's natural plaque removal mechanisms -- macrophage-mediated cholesterol efflux, hepatic clearance, plaque component remodeling, and statin-driven regression -- operate continuously at a baseline rate that, in patients with active disease, is exceeded by the rate of new accumulation. The redistribution mechanism produced by chamber intervention shifts the equilibrium: by providing preferential redeposition sites for mobilized material, the chamber reduces the net new deposition at inaccessible sites, allowing the natural removal mechanisms to operate against a smaller incoming load. Over multiple chamber cycles, coupled with continuing systemic therapy, this provides indirect relief to inaccessible regions of the vasculature, with a cumulative reduction in body-wide plaque burden that exceeds direct chamber extraction alone.

The mechanism does not constitute a cure. Calcified deposits in small vessels are not mobilized by current systemic therapy and remain in place. Microvascular structural remodeling persists despite plaque mobilization. Underlying drivers of atherogenesis (lipid metabolism, inflammatory state, endothelial dysfunction, and vessel aging) continue to operate. The architecture provides a maintenance technology for vascular health rather than a one-time correction; iterative chamber sessions over decades, combined with continuing systemic therapy and lifestyle management, support a sustained reduction in clinically dangerous plaque burden across the patient's lifetime.

Stage-dependent pressure regime

The protocol in Toli specifies a uniform rule: the treatment circuit pressure must be below the bypass circuit pressure at all times [1]. Any leak at the balloon seal is therefore inward (blood diluting the chamber, no systemic chemical exposure). This is correct but uniform. A stage-dependent regime is safer.

During active chemical stages (EDTA, surfactant, and collagenase), treatment circuit pressure is held below bypass pressure, so any leak pulls blood into the chamber (dilutes treatment, no systemic risk from chemicals). During saline-only phases (pre-fill, inter-stage rinse, and post-flush), treatment circuit pressure is held above bypass pressure, so any leak pushes saline into the blood, which is biologically trivial at the volumes involved (0.9% saline at small volumes). The final post-flush is outward pressurized to sweep any residual chemical traces out of the chamber before balloon deflation. The pressure differential during this phase is bounded; a typical operating range would be 1.2-1.5× bypass pressure, sustained for ≤30 seconds. Differentials above approximately 1.5× approach the range associated with endothelial denudation in balloon-injury models [9] and are therefore avoided.

Inward bias is required only during active chemistry. The final outward-biased flush adds an active sweep mechanism not available in the uniform regime.

Direct percutaneous needle access for deep vessels

The topological isolation principle generalizes to any access method that can place balloon occlusion at two points along a vessel. The architecture in Toli assumed catheter access through the peripheral vasculature (femoral and radial), navigating the vascular tree to the target [1]. Direct percutaneous needle access through overlying tissue, at anatomically chosen body surface sites, reaches deep vessels that are difficult or impossible to access through conventional catheter routes.

What this enables

Catheter-based access limits treatment to patients whose lesions are reachable through peripheral arterial anatomy. Direct needle access reaches patients with critical lesions in deep vessels currently inaccessible to routine intervention: renal artery stenosis, mesenteric atherosclerosis, and deep visceral disease. The preventive maintenance population expands to subclinical disease in these deep sites.

The architectural extension preserves all elements of the chamber protocol: balloon occlusion at two points, drainage of blood, saline chamber, treatment circuit, bypass circuit for distal perfusion, sentinel chemistry for leak detection, and stage-dependent pressure regime. The change is only in how the catheter ends arrive at the target vessel: through overlying tissue at body surface entry points rather than through the vascular tree.

Geometry

**Figure 2:**
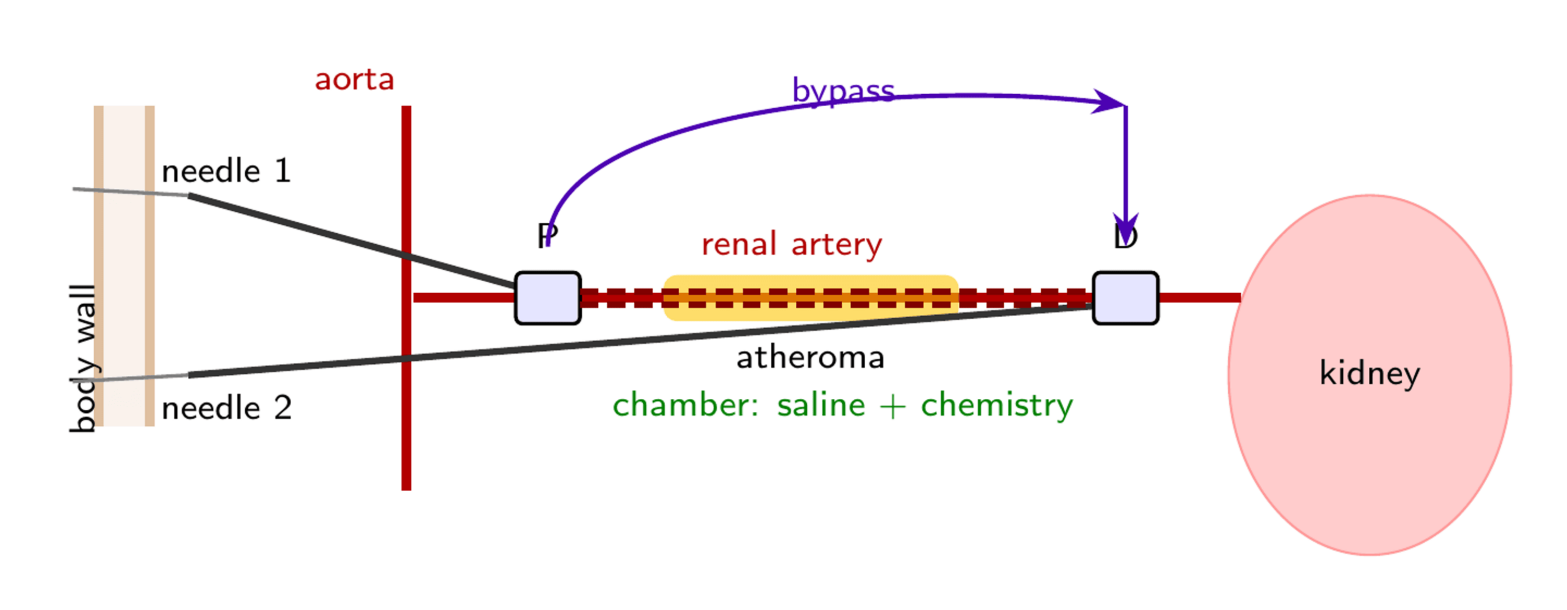
Direct percutaneous needle access geometry for the renal artery, with two needles entering through the flank to isolate a target segment between proximal and distal balloons Figure created with TikZ/PGF (LaTeX)

Two needles, each carrying a balloon catheter, are inserted through overlying tissue at anatomically chosen body surface sites converging on the target vessel. Each needle's path is short and deep rather than long and tortuous: the renal arteries are reached posteriorly through the flank, the mesenteric arteries anteriorly through the abdomen, and the aorto-iliac segments anteriorly through the lower abdomen. Image guidance (ultrasound, CT, fluoroscopy, or cone-beam CT) confirms vessel entry. Inflation isolates a vessel segment between the two needle tips; the segment becomes the saline chamber. The bypass circuit runs externally between the two needles, providing distal perfusion through the same vessel that is being treated. The treatment circuit operates on the isolated segment. The geometry for a renal artery procedure is shown in Figure 2.

The length of the segment is determined by the needle separation, which the operator chooses based on the lesion and the vessel anatomy. For short lesions, the needles can be inserted close together. For longer lesions, the needles are placed farther apart. The geometry is more flexible than catheter-based access because the needle tip positions are decoupled from upstream anatomy.

Existing techniques

Interventional radiology routinely performs direct percutaneous vascular access for related procedures: transjugular intrahepatic portosystemic shunt placement via transhepatic portal vein access, direct puncture of renal veins for adrenal vein sampling, percutaneous biopsy of kidneys and adrenals, vertebroplasty, and percutaneous abscess drainage. All required components are clinically available: introducer sheaths with hemostatic valves, image guidance (ultrasound, CT, fluoroscopy, and cone-beam CT), and puncture closure (manual pressure for small-caliber and Angio-Seal/Perclose for larger).

Worked example: renal artery

A representative renal artery procedure proceeds as follows. The patient is positioned for flank access, with image guidance established before any needle puncture. The first needle is inserted posteriorly through the flank to reach the proximal renal artery near the aorta, with ultrasound-guided vessel entry. A second needle is inserted using a similar approach to reach the distal renal artery near the kidney hilum. Each needle carries a catheter terminating in a balloon, and once both balloons are positioned, simultaneous inflation isolates the target segment between them. A bypass circuit is then established between the two needles, with the external loop maintaining kidney perfusion throughout the procedure. The treatment circuit operates within the isolated segment, and the chamber protocol proceeds through its sequence of stages. After treatment is complete, the chamber is drained, the balloons deflate, and the catheters are withdrawn. A vascular closure device is deployed through each needle path before the needle itself is removed. Final imaging confirms hemostasis at both puncture sites.

A note on hemostasis is in order, and the framing in this paragraph deserves more weight than the worked example procedure description alone conveys. The renal artery operates at systemic pressure and lies in the retroperitoneum, where any bleeding accumulates in a space that does not externally compress and may remain occult until hemodynamic instability develops. Manual compression, the standard closure technique for venous and small-caliber peripheral arterial punctures, is inadequate for direct renal artery puncture at the catheter sizes (5-7 Fr) that the protocol requires. A vascular closure device deployed through the needle path before withdrawal (analogous to Angio-Seal, Perclose, or similar) is the realistic closure technique, but closure device performance in direct percutaneous renal artery access has not been characterized at scale and is itself one of the validation items, not a solved problem.

The validation experiment for needle access (testable prediction 5) must therefore be powered for a safety endpoint, not just for completion rate. A study designed around n≥6 procedures will demonstrate technical feasibility in expert hands but will not exclude clinically meaningful bleeding rates: detecting a 5% retroperitoneal hematoma rate with statistical confidence requires sample sizes an order of magnitude larger. The realistic validation pathway for this refinement therefore proceeds in two stages: a small initial series to demonstrate technical feasibility and to identify failure modes, followed by a substantially larger series powered for safety endpoints (objective bleeding metrics: blood loss, hematoma volume on post-procedure imaging, AND need for transfusion or surgical repair) before any human application is contemplated. Backup strategies for closure failure (proximal balloon tamponade and embolization of the puncture tract) must be predefined as part of the procedural protocol.

Broader applicability of the needle access pattern

The needle access pattern generalizes beyond the renal arteries. The human body is nowhere too thick for needles of appropriate gauge to reach a major blood vessel, with needle paths chosen to avoid sensitive structures and minimize tissue traversal. Direct percutaneous arterial access through carefully selected entry points and trajectories is, all considered, a minor injury for the bargain it offers: reach to deep vessels currently inaccessible to catheter-based intervention, with two small puncture wounds as the access cost. Modern interventional radiology already exploits this principle for percutaneous biopsy, abscess drainage, vertebroplasty, and various transhepatic procedures; minimally invasive surgery exploits it for laparoscopic appendectomy, cholecystectomy, and a wide range of abdominal interventions where needle and trocar entry replaces an open incision. The chamber architecture extends the same pattern to the arterial side. Once the validation experience exists for renal artery access, similar protocols can be developed for mesenteric, hepatic, splenic, and other deep visceral arteries, with entry points and trajectories optimized per anatomy. The pudendal arterial system, accessible via perineal puncture, represents another candidate application: vasculogenic erectile dysfunction driven by proximal atherosclerotic disease in the pudendal arteries could potentially be addressed by chamber-based cleaning at this site, though the smaller vessel caliber (approximately 3-4 mm) places this application at the lower boundary of the architecture's mechanical scope and would require its own validation. The patient population reachable through this approach is substantially larger than the population currently accessible through the peripheral catheter approach.

Organ as chamber: a parallel architectural primitive

A related architectural primitive worth recording, distinct from the segment-isolation architecture developed in this paper, is organ-scale isolation in organs with discrete arterial inflow and discrete venous outflow. Rather than isolating a segment between two balloons in the same vessel, occlusion is placed at the organ's arterial inflow and at the organ's venous outflow. The entire organ becomes the chamber. Chemistry perfuses the organ's full vascular tree, including microvasculature inaccessible to segment-style isolation, with effluent collection at the venous side.

Candidate organs are those with a relatively concentrated arterial and venous supply, modest metabolic demand, and tolerance to short ischemic windows. The penis, with the pudendal arterial system as inflow and the dorsal venous system as outflow, is one example; the cavernosal microvasculature that drives vasculogenic erectile dysfunction would be reached by chamber chemistry through this organ-scale isolation rather than by direct cavernosal access. Other candidates include the thyroid, the testis, individual organs, or organ portions whose vasculature can be isolated at the organ-vascular interface. Each application requires anatomical specification of the inflow and outflow occlusion sites, ischemic tolerance characterization for the organ, and consideration of venous occlusion safety. These are open architectural and validation items, recorded here as a future direction parallel to the vessel-segment chamber rather than as developed protocols.

Clinical implications

The patient population currently treated for atherosclerotic disease through percutaneous methods is a small fraction of those with clinically significant atherosclerotic disease. The remainder includes patients whose disease is concentrated at deep vessels (renal, mesenteric, and retroperitoneal) where catheter-based access is impractical, patients whose lesions are at locations along major vessels where catheter access is feasible but unfavorable, and patients with subclinical or preventive-maintenance indications where the threshold for catheter-based intervention is too high to justify the systemic exposure. Direct needle access addresses each of these.

Limitations of the needle access approach

The needle access approach inherits all the limitations of the chamber protocol (seal integrity, ischemic tolerance, and calibration uncertainty in the first cycle) and adds the direct access risks: bleeding at puncture sites, vascular injury during needle advancement, and infection along needle tracts. These risks are not novel in interventional medicine, but the safety profile of this specific configuration must be established empirically. Vessels with severely calcified or fibrotic walls may not seal adequately around an inflated balloon delivered through a needle; these are the same vessels that are challenging for catheter-based balloon seal. Vessels surrounded by sensitive structures (the renal artery's proximity to the renal vein and the mesenteric arteries' proximity to the bowel) require careful trajectory planning. Operator training requirements for direct arterial puncture under image guidance are non-trivial.

Post-cleaning biodegradable protective coating

Cleaning exposes a vulnerable surface. Intact endothelium is removed or absent across much of the cleaned region. Dead and dying tissue within the plaque is exposed at the plaque wall boundary. The denuded surface is thrombogenic, inflammatory, and susceptible to renewed plaque initiation until re-endothelialization completes. In porcine balloon injury models, re-endothelialization of mechanically denuded vessel surfaces proceeds over the order of weeks [9]. Patient factors that impair endothelial recovery (diabetes, chronic kidney disease, advanced age, and longstanding endothelial dysfunction) extend this timeline. The protective coating must persist across the relevant window, which means the dissolution kinetics should be tunable to the expected patient biology rather than fixed at a single timescale.

The protocol in Toli accommodates this healing timeline implicitly through its iterative session and interval structure but relies on systemic antithrombotic therapy to manage the vulnerable window [1]. A localized biodegradable protective coating applied to the cleaned surface at the end of each session addresses the window directly rather than through systemic drug exposure.

Mechanism

After the final saline flush, with active chemistry and mechanical cleaning both completed and the chamber still isolated and filled with saline, a coating precursor is introduced and allowed to polymerize or gel in situ against the cleaned vessel wall. Balloons deflate; blood returns to flow over the coating rather than over the bare denuded surface.

The coating serves two functions during the healing window: an antithrombotic surface preventing acute clot formation on exposed subendothelial tissue, and a physical barrier preventing blood contact with dead and dying tissue at the plaque wall boundary. It dissolves on a timescale matched to re-endothelialization, leaving no permanent foreign material in the vessel.

Compared with current post-atherectomy practice, which bridges the re-endothelialization window with systemic dual antiplatelet therapy for weeks to months and accepts the bleeding risk that accompanies it, a localized biodegradable coating delivers protection at a higher effective concentration at the site, reduces systemic drug burden, and matches the dissolution timescale to expected healing biology. Systemic antithrombotic therapy may still be warranted but can be shorter or lighter.

Candidate coating materials

Biodegradable vascular coatings are an established research area. Candidate materials include polylactic acid/polyglycolic acid copolymers (well-characterized degradation kinetics, used in biodegradable stent scaffolds, tunable by composition ratio), hyaluronic acid (biocompatible, hydrophilic, dissolves over days to weeks), hydrogels with engineered crosslink density, and drug-eluting biodegradable matrices that release antithrombotic or pro-healing agents as the matrix erodes. The specific choice depends on which timescale matches re-endothelialization in the target population, which secondary functions are wanted, and which deployment chemistry is compatible with the post-cleaning chamber environment.

Graded thickness matched to healing geometry

Re-endothelialization does not proceed uniformly. Endothelial cells migrate inward from the edges of the cleaned region, so the perimeter heals first and the center heals last. The geometry has two relevant directions, axial (along the vessel) and circumferential (around the vessel circumference), and the gradient acts differently along each.

Along the axial direction, for cleaned segments long enough that the segment length is greater than several characteristic endothelial migration distances, the coating gradient should be axial: thinner near the segment ends (which the endothelium reaches first from the adjacent intact vessel) and thicker near the segment center (which the endothelium reaches last).

Along the circumferential direction, the entire annulus around any axial position is denuded simultaneously and re-endothelializes primarily from its axial neighbors, not from circumferential neighbors. There is no large circumferential gradient in healing time at a given axial position. The coating thickness should therefore be approximately uniform circumferentially at each axial position and should be graded along the axis.

For short, cleaned segments where the segment length is comparable to or smaller than the characteristic migration distance, the entire segment heals on a similar timescale and a uniform thickness coating suffices. The graded approach is most useful for long segments where the central portions of the cleaned region are far from an intact endothelial source.

Engineering of graded coatings

For in-situ deposition during the cleaning protocol, achieving graded thickness can be approached through multistep deposition (a thin layer applied across the cleaned region first, additional layers concentrated in the center through controlled flow patterns), through a concentration gradient during deposition (precursor introduced at chamber center and withdrawn at edges), through edge-biased dissolution enhancement (uniform initial coating followed by brief exposure to an agent that accelerates dissolution at the edges), or through any other engineered gradient in polymerization conditions across the chamber. Which method is optimal depends on the chosen coating chemistry and is an empirical engineering question for validation.

Secondary diagnostic dimension

The dissolution pattern becomes observable through post-procedure imaging (ultrasound, OCT, and angiography). Coating persisting longer than expected in specific regions indicates delayed healing at those locations, which could prompt additional clinical management. The coating's disappearance timeline serves as an indirect indicator of healing progress, with the same per-cycle effluent signals that drove the cleaning decisions during treatment now coupled to a post-treatment monitoring channel. Treatment becomes diagnostic, and diagnostic information feeds into clinical management beyond the procedure itself.

Future directions

Several directions extend the architecture beyond what is specified here. They are noted for future researchers, not developed as protocols ready for validation.

Expanded mechanical methods within the chamber

The pointed balloon with pressure feedback is recommended as the first-generation mechanical tool for reasons of regulatory shortness and clinician familiarity. The chamber, however, expands the operational envelope of every percutaneous mechanical method, and future operators may exploit this in several ways. Even cartilaginous or moderately cohesive atheroma can be reduced mechanically when the embolic constraint is removed, allowing methods more aggressive than balloon contact: rotational atherectomy burs, orbital atherectomy crowns, directional cutters, or scoring and cutting balloons can all be applied within the chamber to substrates they could not safely engage in flowing blood. A scoring balloon used early in the session to score the fibrous cap longitudinally can release softer underlying material into the next chemistry stage, potentially shortening that stage substantially; whether to do this depends on lesion characterization at the start of the session. Balloon surface modifications beyond the smooth or scoring profiles in current clinical use, including textured or abrasive surfaces with experimentally determined coarseness graded across balloon sizes, are open engineering directions. The role of these methods in the primary protocol depends on validation studies that have not been performed; the architecture supports their use if and when those studies establish appropriate safety and efficacy bounds.

Anatomical extensions

The architecture is not specific to long vessel segments. Two anatomical contexts that may eventually accept the topological isolation principle are noted here, with the caveat that both face structural difficulties that would have to be resolved before either could be developed into a procedure.

Coronary ostial cleaning

Atheromatous disease at the coronary ostia is clinically important because ostial lesions affect all downstream coronary flow, and conventional catheter access to the ostia is difficult. A retrograde approach from peripheral arterial access through the aorta to the aortic root, with isolation of the specific ostium being treated, is geometrically appealing.

The geometric problem to solve is the seal. The aortic root is large, pulsatile, and the coronary ostia open into curved sinuses (the sinuses of Valsalva). A balloon inflated in the root above an ostium would occlude the entire root, including the contralateral coronary, not just the target ostium. A balloon designed to seal around an ostium against its sinus wall is a different device from the symmetric occlusion balloon used for vessel segment cleaning, and it is not currently in the protocol's hardware set. A workable design would likely be a side-engaging balloon with an asymmetric inflation profile and a soft circumferential rim that conforms to the sinus geometry. Whether such a device can be built with adequate sealing and atraumatic engagement is an open engineering question. The retrograde access path itself is straightforward; the difficulty is local to the seal.

If the seal problem is solved, the ostial protocol would complement rather than compete with the external coronary cleaning in Toli [1]. The protocol in Toli addresses lesions along the length of the coronary artery; the ostial protocol would address the distinct lesion population at the vessel origin [1].

Aortic valve cleaning

Calcific aortic stenosis is among the most common elderly cardiac conditions, and a chemistry-based restoration of native leaflet function would be transformative if achievable. The architecture would isolate the valve plane between two balloons (one in the aortic root, one in the left ventricular outflow tract) and apply the combined protocol locally to the leaflets.

Three difficulties stack against this application and warrant honest acknowledgment. The first is matrix scale. Vessel walls are millimeter-thick and have redundant structural elements; the medial layer can tolerate some matrix damage during chemistry exposure because the wall as a whole has reserve mechanical capacity. Valve leaflets are tens to a few hundred microns thick, and the matrix mechanics are the leaflet's mechanical function. There is no margin for matrix damage as there is in a vessel wall. The cellular biology paragraph above understates this asymmetry: the question is not whether valve interstitial cells tolerate EDTA exposure but whether the leaflet matrix retains its mechanical integrity after chemistry, and any damage to that matrix is functionally consequential.

The second is the time budget. Cardiac output reduction during aortic valve occlusion limits the cleaning window to seconds or a few minutes per session. EDTA dissolution rates of valvular hydroxyapatite at clinically tolerable temperatures, by every plausible estimate, would require many such windows distributed across many separate sessions, with full circulation restored between each. The protocol becomes a sequence of short chemistry pulses with full circulation in between, which is operationally different from the vessel segment protocol and would require its own design.

The third is dystrophic versus atherosclerotic calcification chemistry. The hydroxyapatite crystalline endpoint is similar in the two contexts [10,11], but valve calcification arises from osteogenic differentiation of valve interstitial cells [11] in a matrix dominated by collagen and proteoglycans, while atheromatous calcium forms through smooth muscle cell apoptosis in a collagen and lipid matrix. Chelator access to crystal surfaces and the kinetics of dissolution may differ in ways that have not been characterized.

For these reasons, the valve application is recorded here as a future direction. Validation would begin with explanted calcified human or porcine aortic valves, characterizing dissolution kinetics and matrix integrity under chamber-equivalent EDTA exposure before any in-vivo work. A separate paper devoted to that work, with the experimental data in hand, is the right form for this idea once the data exist.

Quantitative modeling

A quantitative model of the mechanical-chemical interaction dynamics, calibrated against porcine validation data, would predict how mechanical pre-cleaning shifts the substrate state for subsequent chemistry stages, how per-stage extraction yields combine into total extraction, and how the protocol's convergence depends on lesion morphology. Such a model is a prerequisite for clinical trial design and is the natural follow-on subject after the porcine experimental data exist.

## Discussion

Testable predictions

Validation uses the porcine atherosclerosis model established in a study by Toli [1]. Atheromatous lesions are induced in cholesterol-fed Yucatan or Ossabaw miniature swine over 6-12 weeks of high-cholesterol diet, supplemented by mechanical injury through balloon endothelial denudation following the standard porcine restenosis protocol [9]. Vessel calibers appropriate to the architecture include the porcine left anterior descending coronary artery (approximately 3 mm diameter), the renal artery (approximately 4-5 mm), and the iliac artery (approximately 5-6 mm). Endpoints combine real-time IVUS and OCT within the saline-filled chamber, post-procedure histology with Movat pentachrome staining for plaque component identification, and mass collection from the effluent for direct quantification of removed material. The hypothesis generates five experimentally testable predictions.

First, the weakly adhered fraction of plaque mass on a typical lesion can be quantified by gentle mechanical removal in the isolated chamber. Quantification by mass collection of effluent debris from the mechanical pre-cleaning stage, normalized by total plaque mass on the same lesion (assayed at experimental endpoint by histology). The hypothesis is supported if the weakly adhered fraction falls in the 40%-60% range across a representative population of lesion morphologies, with the median and interquartile range to be reported. The hypothesis is contradicted if the fraction is consistently below 20% (chemistry-only suffices) or consistently above 80% (chemistry is largely unnecessary).

Second, mechanical pre-cleaning followed by reduced chemistry achieves more complete plaque removal than chemistry alone. Quantification by post-treatment plaque mass (IVUS and histology) was compared between matched lesions treated with the chemistry-only protocol and the combined protocol. The hypothesis is supported if matched lesions show greater plaque mass reduction in the combined protocol with appropriate statistical significance.

Third, the combined protocol allows substantially reduced collagenase exposure relative to the chemistry-only protocol for equivalent plaque removal. Quantification by titrating the collagenase dose and exposure time downward in the combined protocol until plaque removal endpoints match the chemistry-only protocol's results. The hypothesis is supported if matched removal is achieved at or below 60% of the chemistry-only collagenase burden. This 60% threshold corresponds to the structurally integrated fraction of plaque mass under the two-regime model: with the weakly adhered fraction removed mechanically, only the structurally integrated fraction (at most 60%) remains for chemistry to address. Achievement materially below this threshold would constitute empirical evidence for additional exposed-substrate kinetic enhancement.

Fourth, the localized biodegradable coating reduces post-procedure thrombotic and inflammatory events relative to systemic antithrombotic therapy alone. Quantification in a longitudinal animal study following coated and uncoated treated segments at 1, 4, 8, and 12 weeks post-procedure, with thrombus formation, neointimal hyperplasia, and inflammatory infiltrate as primary endpoints assayed by histology and imaging.

Fifth, direct percutaneous needle access to a deep vessel (renal artery) achieves complete topological isolation and successful protocol execution comparable to catheter-based access of an accessible vessel. Quantification by completeness of effluent recovery, absence of leak detected by sentinel chemistry, and successful procedure completion in n≥6 porcine renal artery cleaning procedures. The hypothesis is supported if needle access procedures match catheter access procedures in completion rate and effluent quality.

Limitations

The work is at the concept stage; the limitations below warrant explicit acknowledgment.

(a) The two-regime plaque model is supported by the structural inhomogeneity observed in pathological specimens, but the quantitative partitioning between regimes (the 40%-60% weakly adhered estimate) is empirical and lesion-dependent. The actual partition across a clinical population is testable prediction 1. (b) The pointed balloon with pressure feedback approach assumes that gentle mechanical contact distinguishes weakly adhered material from structurally integrated material with adequate spatial resolution. This is a hypothesis based on the qualitative mechanics of layered atheroma; quantitative validation in real lesions across a representative population requires the porcine experiments. (c) The pressure feedback signal is an integrated measure of resistance over the full balloon-vessel contact area. Focal high-stress inclusions (small calcified spikes within otherwise soft material) may not raise the integrated signal above threshold despite producing dangerous local stress on intima or media. Conservative threshold calibration mitigates this; supplementary intra-chamber imaging recovers the missing channels for cases where focal inclusions matter. (d) The pressure feedback threshold calibration cycle has been specified but not validated. The asymmetric ratchet (initialize at population ceiling, adjust only downward) is a safety measure rather than a derivation; the appropriate population ceiling for each vessel class and indication is itself an empirical question. (e) The residual collagenase burden under the combined protocol is bounded above by the structurally integrated fraction of plaque (40%-60% of the chemistry-only specification) and may be lower if exposed-substrate kinetics provide an additional efficiency multiplier. The magnitude of any such kinetic enhancement is not derived in this work; the actual residual achievable while preserving cleaning efficacy is empirical and is testable prediction 3.

(f) Direct percutaneous needle access to deep vessels has been described in the protocol, but the closure device performance for renal-artery-sized punctures (5-7 Fr) at this location has not been characterized at scale. Vascular closure device performance is itself one of the validation items. (g) The biodegradable coating concept is well supported by existing biomaterials research, but the specific deployment chemistry (in-situ polymerization in a saline-filled chamber, against a denuded vessel surface, with potential post-cleaning chemical residue) requires preclinical material screening before in-vivo work. Coating adhesion to denuded vessel walls under returning pulsatile flow, dissolution kinetics tuned to expected re-endothelialization windows, and cytotoxicity profiles are open questions. (h) The coronary ostial application requires a side-engaging asymmetric balloon that is not currently in the protocol's hardware set. The seal geometry against the sinus of Valsalva is an open engineering question. (i) The aortic valve application assumes EDTA dissolves valve calcification effectively. Dystrophic valve calcification has different matrix chemistry from atherosclerotic plaque calcification; the assumption requires empirical validation before clinical application. (j) The combined protocol's session duration depends on the lesion: in lesions where the bulk fraction is removed quickly by mechanical pre-cleaning, the combined session can run faster than the chemistry-only baseline; in lesions where mechanical pre-cleaning is slow, the session may be longer than chemistry-only and may push against the ischemic tolerance of territories supplied by minor branches within the isolated segment. (k) A quantitative model of the mechanical-chemical interaction dynamics is not developed here. Such a model would predict how mechanical pre-cleaning shifts the substrate state for subsequent chemistry stages, and how the per-stage yields combine into total extraction. It is a prerequisite for clinical trial design and is left to follow-on work informed by the porcine experiments. (l) The EDTA stage endpoint specified as "effluent Ca²⁺ returns to baseline" is confounded by inward leak of blood Ca²⁺ (approximately 1.2 mM ionized) at the balloon seal under any seal compromise. The endpoint must therefore be interpreted as effluent Ca²⁺ returning to a baseline that subtracts pre-procedure input saline Ca²⁺ (near zero) and accounts for any sentinel-detected blood admixture. The same caveat applies to a lesser degree to other effluent channels, but Ca²⁺ is the most affected because blood Ca²⁺ is non-negligible.

(m) The general parameter freedom argument is asserted on architectural grounds; the optimal combinations of chemistry concentration, treatment temperature, flow rate, and stage duration for specific lesion morphologies have not been mapped and require systematic validation. (n) The hemodynamic redistribution synergy with systemic therapy is mechanistically plausible but unvalidated. The magnitude of preferential redeposition at chamber-cleaned segments and the cumulative reduction in body-wide plaque burden across multiple chamber cycles require longitudinal data outside the current validation plan. (o) The broader needle access applicability beyond renal arteries is architectural; validation in this work is renal-artery-specific, and each additional vessel class (mesenteric, hepatic, splenic, and pudendal) requires independent characterization of access trajectory, closure performance, and adjacent-structure injury risk. (p) The organ as chamber primitive is recorded as a parallel architectural primitive; no specific procedure has been developed or validated. Ischemic tolerance for organ-scale occlusion, anatomical specification of arterial inflow and venous outflow occlusion sites, and venous-occlusion safety profile are open items for each candidate organ.

## Conclusions

The topologically isolated arterial segment supports a richer treatment protocol than chemistry alone. Mechanical pre-cleaning of the weakly adhered plaque fraction by a pointed balloon with pressure feedback reduces the collagenase burden of the chemistry-only protocol. During chemical stages, the pressure differential biases inward; during saline phases, it reverses outward -- safer than uniform inward bias. Direct percutaneous needle access extends the architecture to deep vessels currently inaccessible to catheter-based intervention. A localized biodegradable protective coating bridges the re-endothelialization window with local rather than systemic antithrombotic protection. Five testable predictions provide a path to porcine validation.
